# Antifungal and Antioxidant Activity of Thiourea Derivatives Against Nosocomial *Candida auris* Strains Isolated in Romania

**DOI:** 10.3390/molecules30081675

**Published:** 2025-04-09

**Authors:** Marina Ionela Nedea (Ilie), Carmellina Daniela Bădiceanu, Irina Gheorghe-Barbu, Ioana Cristina Marinaș, Radu Pericleanu, Rareș-Ionuț Dragomir, Andreea Ștefania Dumbravă, Ana Maria Dascălu, Dragoș Șerban, Corneliu Tudor, Madălina Solomon (Preda), Oana Popescu, Andreea Letiția Arsene, Bruno Ștefan Velescu

**Affiliations:** 1 Faculty of Pharmacy, “Carol Davila” University of Medicine and Pharmacy, 6 Traian Vuia Street, 020956 Bucharest, Romania; marina.nedea@umfcd.ro (M.I.N.); carmellina.badiceanu@umfcd.ro (C.D.B.); andreea.arsene@umfcd.ro (A.L.A.); bruno.velescu@umfcd.ro (B.Ș.V.); 2 Faculty of Biology, University of Bucharest, Intrarea Portocalelor No. 1-3, 060101 Bucharest, Romania; pericleanuradu@gmail.com (R.P.); r.dragomir20@s.bio.unibuc.ro (R.-I.D.); andreeadum29@gmail.com (A.Ș.D.); 3 The Research Institute of the University of Bucharest (ICUB), 050095 Bucharest, Romania; 4 Faculty of Medicine, “Carol Davila” University of Medicine and Pharmacy, 020021 Bucharest, Romania; ana.dascalu@umfcd.ro (A.M.D.); dragos.serban@umfcd.ro (D.Ș.); corneliu.tudor@umfcd.ro (C.T.); 5 Emergency University Hospital Bucharest, 050098 Bucharest, Romania; 6 Department of Microbiology, Parasitology and Virology, Faculty of Midwives and Nursing, “Carol Davila” University of Medicine and Pharmacy, 020021 Bucharest, Romania; madalina.preda@umfcd.ro; 7 Marius Nasta Institute of Pneumology, 050159 Bucharest, Romania; oana.popescu@marius-nasta.ro

**Keywords:** *Candida auris*, antioxidants, thiourea derivatives, antihemolytic

## Abstract

Nosocomial fungal infections caused by *Candida auris* pose a threat to public health due to their increased resistance to common antifungal drugs. Four thiourea derivatives of 2-thiophenecarboxylic acid were evaluated for their antifungal and antioxidant activity. The antifungal activity of the compounds was tested against strains of *C. auris* isolated from a hospital in Romania. With a notable inhibitory effect on *C. auris* biofilm growth and microbial adherence, the ortho-methylated derivative (SB2) showed the highest antifungal activity. Furthermore, emphasizing the impact of structural factors on the electron-donating capacity of these compounds, antioxidant activity assays (DPPH, FRAP, TEAC and CUPRAC) identified the SB2 compound as having the highest antihemolytic and antioxidant effects. The low cytotoxicity validated by hemocompatibility assays makes these compounds options for antifungal treatment. The results show that antifungal and antioxidant action is greatly influenced by structural modifications, especially the position of the methyl group on the aromatic ring. The possible clinical uses of these molecules as drugs for the treatment of multidrug-resistant *C. auris* infections needs further investigation.

## 1. Introduction

Nosocomial fungal infections represent a growing global public health threat, with *Candida auris* emerging as a particularly concerning pathogen due to its multidrug resistance and high associated mortality. First identified in 2009 in Asia, *C. auris* has rapidly disseminated across continents, displaying unique phenotypic and genotypic characteristics that complicate both diagnosis and treatment [1,2,3].

Unlike other *Candida* species that typically colonize mucosal surfaces such as the gastrointestinal tract, *C. auris* shows a predilection for colonizing the skin, thereby facilitating direct person-to-person transmission, particularly in healthcare environments [4]. Additionally, *C. auris* is capable of causing invasive infections such as candidemia, which is frequently associated with mortality rates ranging from 30% to 70%. The pathogen’s persistence on surfaces and resistance to common disinfectants have contributed to recurrent healthcare-associated outbreaks, especially among immunocompromised or critically ill patients [5].

The global spread of *C. auris* is attributed to several factors: efficient patient-to-patient transmission, environmental persistence, and widespread resistance to major antifungal drug classes. The increasing incidence of resistance to triazoles, polyenes, and echinocandins has led to the emergence of pan-resistant strains, particularly in outbreak settings such as those reported in the United States [6,7,8,9]. Compared to non-*auris Candida* infections, the multidrug-resistant nature of *C. auris* substantially limits therapeutic options and contributes to worse clinical outcomes.

The virulence of *Candida* spp., including *C. auris*, is mediated by several factors, such as the secretion of hydrolytic enzymes (e.g., proteases and lipases), the expression of mannosyl transferases, oligopeptide transport systems, siderophore-mediated iron acquisition, and the ability to form biofilms. These traits facilitate host colonization, tissue invasion, immune evasion, and nutrient acquisition, collectively enhancing pathogenicity [6].

A major clinical challenge associated with *C. auris* is its frequent misidentification by conventional phenotypic and biochemical methods including VITEK 2, API 20C AUX, and BD Phoenix systems, which often misclassify it as *Candida haemulonii*, *Candida famata*, or *Candida lusitaniae.* Such diagnostic inaccuracy can delay the initiation of appropriate antifungal therapy, which is particularly concerning given *C. auris*’s multidrug resistance to azoles, polyenes, and echinocandins. Such delays contribute to therapeutic failure, prolonged infection, increased transmission risk, and elevated patient morbidity and mortality. To address these limitations, advanced diagnostic tools have been validated for their high sensitivity and specificity in detecting *C. auris* directly from clinical specimens. These include matrix-assisted laser desorption/ionization-time of flight (MALDI-TOF) mass spectrometry, polymerase chain reaction (PCR) assays targeting the internal transcribed spacer (ITS) and D1/D2 regions of the 28S rDNA, real-time PCR, and whole-genome sequencing (WGS) [10,11,12]. The adoption of these molecular techniques enables timely and accurate identification, facilitating a prompt initiation of the appropriate antifungal therapy and effective infection control measures.

*C. auris* has been reported in at least 47 countries across all inhabited continents, although underreporting due to diagnostic limitations likely underestimates its true prevalence. Genomic studies have identified five major clades—South Asian (clade I), East Asian (clade II), African (clade III), South American (clade IV), and Iranian (clade V)—each with distinct genetic and phenotypic features. Clades I and IV are associated with high antifungal resistance, clade III demonstrates variable susceptibility, while clades II and V are more often linked to colonization rather than invasive disease [10].

In response to these challenges, there is an ongoing investigation into novel antifungal agents. Among the promising candidates are thiourea derivatives, a class of compounds with reported diverse biological activities including antimicrobial, antioxidant, and antifungal properties [12]. Recent studies have demonstrated that certain thiourea derivatives exhibit potent inhibitory activity against *Candida albicans* and *Candida glabrata* strains [13]. Bădiceanu et al. have previously synthesized and characterized thiourea derivatives based on 2-thiophenecarboxylic acid, highlighting their antimicrobial potential and laying the groundwork for further therapeutic exploration [12,14].

Building on previous findings, the present study investigates the antifungal activity and antioxidant capacity of four newly synthesized thiourea derivatives based on 2-thiophenecarboxylic acid, with a specific focus on their efficacy against nosocomial *C. auris* strains. Additionally, this study evaluates the impact of these compounds on biofilm formation, aiming to elucidate their potential as novel therapeutic candidates for the treatment of infections caused by multidrug-resistant fungal pathogens.

## 2. Results and Discussion

### 2.1. Distribution, Antifungal Susceptibility, and Virulence Characteristics of Candida auris Strains

#### 2.1.1. Strain Distribution by Isolation Source

Regarding the source of isolation, three of the eight strains (37.5%) were isolated from urine, due to the use of urinary catheters, which facilitate urinary superinfections. The presence of urinary catheters is known to promote biofilm formation, increasing the risk of *C. auris* colonization and a subsequent infection, with longer catheter use and suboptimal hygiene further amplifying this risk. Implementing stringent infection prevention measures, such as a regular assessment of the necessity of the catheter, adherence to aseptic insertion techniques, and maintaining proper catheter care, can mitigate this risk. Additionally, ensuring patient hygiene, including regular washing of the body with soap and water, can help prevent or control infections [15]. Strains isolated from aspirate were present in the intubated patients, who were unable to breathe independently. Vaginal discharge is a common source of isolation for *Candida* spp. The isolation of *C. auris* strains is common in immunocompromised patients in an intensive care unit suffering from systemic infections. Approximately 67% of *C. auris* isolates are from blood as a result of candidemia [16]. Candidemia caused by *C. auris* is strongly linked to multiple predisposing factors, including the presence of central venous or urinary catheters, underlying immunosuppressive conditions (such as HIV infection, hematologic malignancies, solid organ transplantation, neutropenia, chemotherapy, or long-term corticosteroid use), diabetes mellitus, and chronic kidney disease. In immunocompromised persons, specific clinical and demographic characteristics have been significantly associated with the development of *C. auris* bloodstream infections. These include prolonged stays in intensive care units, pre-existing respiratory conditions, recent vascular surgeries or invasive procedures, and prior exposure to antifungal agents. Furthermore, comorbid chronic kidney disease, colonization with multidrug-resistant organisms, and the extended use of vasopressor support have been identified as additional independent risk factors contributing to the onset of *C. auris* candidemia [17].

#### 2.1.2. Strain Distribution by Susceptibility Profiles to Conventional Antifungals

**Figure 1:**
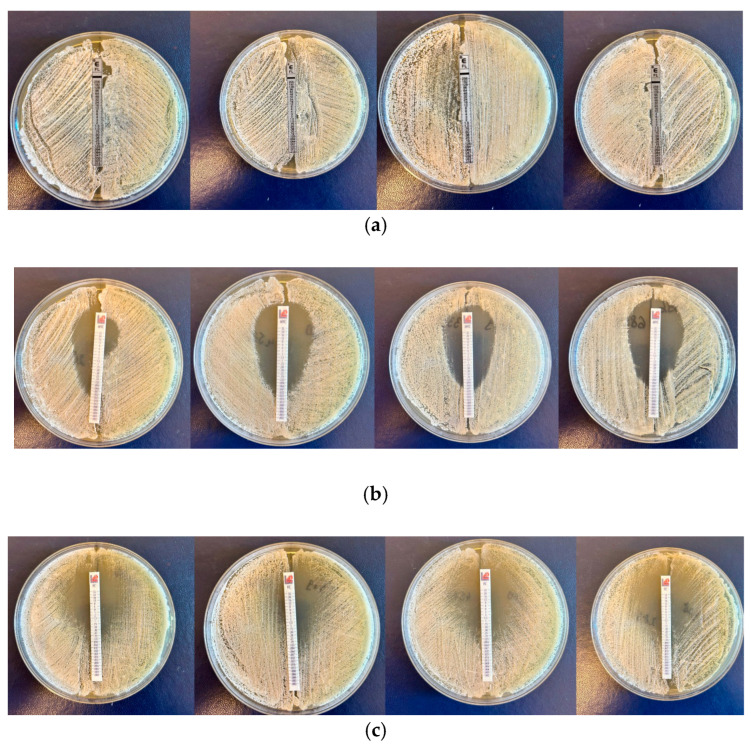

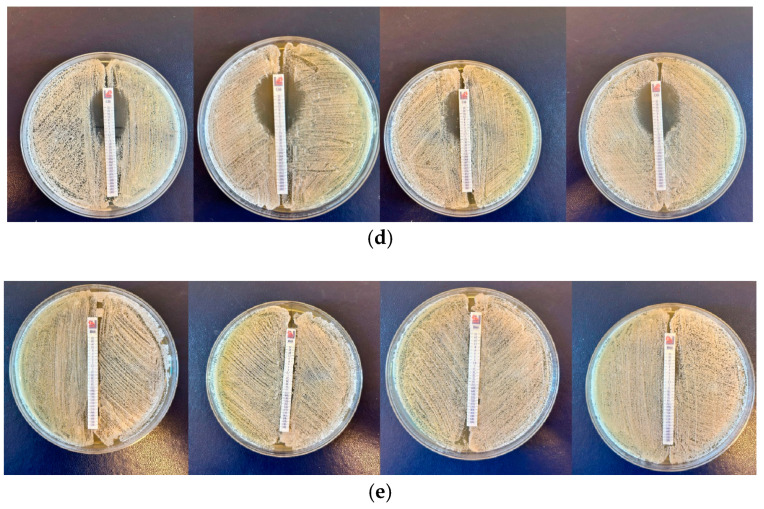
(**a**) Susceptibility of *C. auris* encoded 3396, 1370, 9096, and 6328 strains to fluconazole. (**b**) Susceptibility of *C. auris* encoded 3396, 1370, 9096, and 6328 strains to micafungin. (**c**) Susceptibility of *C. auris* encoded 3396, 1370, 9096, and 6328 strains to flucytosine. (**d**) Susceptibility of *C. auris* encoded 3396, 1370, 9096, and 6328 strains to caspofungin. (**e**) Susceptibility of *C. auris* encoded 3396, 1370, 9096, and 6328 strains to amphotericin B.

In order to interpret the obtained results from susceptibility testing to conventional antifungals, the CLSI standard, 2018 edition M60, was used to establish the resistance/susceptibility profile of the strain to each antifungal tested [18]. The results obtained are described in Table 1 and Figure 1a–e.

According to the obtained results using the E-test method, all eight *C. auris* isolates were resistant to fluconazole and amphotericin B, while for micafungin, caspofungin and flucytosine were susceptible.

Resistance to fluconazole and amphotericin B in *C. auris* is intrinsic, attributable to specific genetic mutations acquired by the species. Fluconazole resistance is primarily associated with mutations in the *ERG11* gene, which encodes the lanosterol 14-α-demethylase enzyme—a key target of azole antifungals. For example, the substitution of tyrosine (Y) with phenylalanine (F) at position 132 (Y132F) leads to a high level of fluconazole resistance in clade I; the substitution of lysine (K) with arginine (R) at position 143 (K143R) leads to fluconazole resistance in clade IV; or F126L (the substitution of phenylalanine with leucine) causes fluconazole and voriconazole resistance in clade II [8,19]. These mutations reduce the enzyme’s binding affinity for azoles, thereby diminishing their efficacy. A high level of amphotericin B resistance is linked to mutations in the *ERG6* gene (for e.g., gene deletion—*ΔERG6*), which alters the sterol composition of the fungal cell membrane, reducing the drug’s ability to bind to ergosterol and disrupting membrane integrity [19,20,21,22].

A study conducted in 2022 by Stanciu et al. included a total of 42 strains of *C. auris* isolated from hospitalized patients in three hospitals in Bucharest, Romania. The obtained results showed resistance to fluconazole and amphotericin B and sensitivities to the other tested antifungals [22]. Fluconazole and amphotericin B are traditional antifungal agents used in the management of invasive fungal infections [23]. Fluconazole inhibits lanosterol 14-α-demethylase (encoded by the *ERG11* gene), a key enzyme in the ergosterol biosynthesis pathway that leads to the accumulation of toxic sterol intermediates with fungistatic effects or the alteration of cell membrane integrity, while amphotericin B binds to membrane ergosterol, causing pores, cell leakage, and a fungicidal effect. However, due to their frequent use, resistance in *C. auris* has become widespread, as also seen in our isolates. By contrast, echinocandins such as micafungin and caspofungin remain the first-line treatment recommended by the CDC and showed consistent efficacy in our study. Flucytosine that is converted intracellularly to 5-fluorouracil disrupts fungal RNA and DNA synthesis, demonstrates preserved activity, and is typically used in combination regimens for invasive candidiasis. Their limited use in Romanian hospitals may contribute to the lower resistance rates observed in our strains [24].

#### 2.1.3. Detection of Soluble Virulence Factors in *C. auris* Strains Using Selective Culture Media

**Figure 2:**
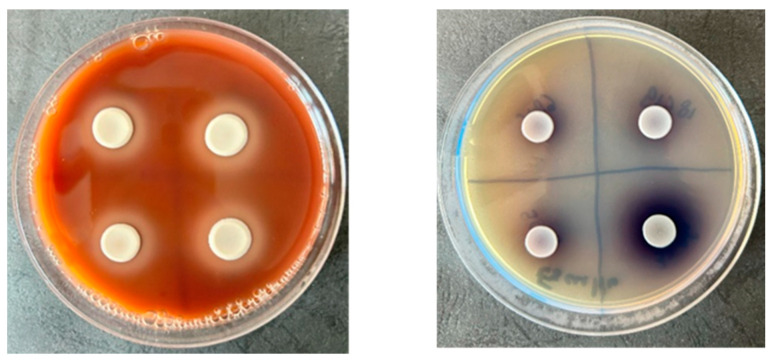
Representative image showing the production of hemolysins and esculin hydrolysis by *C. auris* encoded 3396, 1370, 9096, and 6328 strains on selective culture media.

The soluble virulence factors produced by eight nosocomial *C. auris* isolates are summarized in Table 2 and Figure 2. An evaluation of soluble virulence factor production revealed that all tested strains exhibited β-hemolysin activity and esculin hydrolysis, while no activity was detected for the other investigated virulence factors. Notably, the presence of hemolysins in *C. auris* has been previously correlated with itraconazole resistance, as reported by Jaiswal et al. [25]. These enzymes are critical determinants of *Candida* spp. pathogenicity, facilitating tissue invasion and colonization within the host. In *C. auris*, β-hemolysin production enhances competitive iron acquisition, supporting fungal proliferation and accelerating systemic dissemination [26]. Additionally, *C. auris* strains exhibit esculin hydrolysis, a metabolic trait more commonly associated with cocci. Notably, β-hemolysin-producing strains also demonstrated esculin hydrolysis, suggesting a potential functional correlation between these virulence factors [27].

### 2.2. Biological Assessment of Newly Synthesized Compounds

#### 2.2.1. Antioxidant Activity of Newly Synthesized Compounds

Badiceanu et al. described the synthesis of the four thiourea derivatives, starting from the reaction of 2-thionyl isothiocyanate with aniline and the corresponding aromatic primary amines. The compounds were characterized using IR, ^1^H NMR, and ^13^C NMR spectroscopy, which confirmed their structures. The spectroscopic data indicated the presence of the expected functional groups and showed strong agreement with the proposed molecular structures [12].

**Figure 3:**
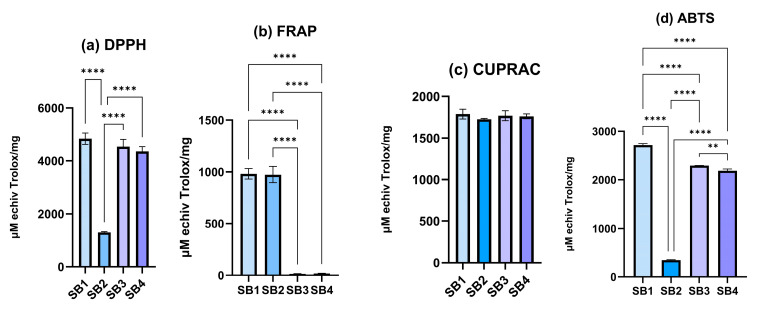
Comparative antioxidant activity between SB1, SB2, SB3, and SB4 obtained through DPPH (**a**), FRAP (**b**), CUPRAC (**c**), and TEAC (**d**) assays. (** *p* < 0.01, **** *p* < 0.0001).

Antioxidant activity was evaluated using four methods to establish the possible mechanism by way of which these four compounds can exhibit this activity. The position of the methyl radicals seems to significantly influence antioxidant activity through the DPPH, FRAP, and TEAC methods. The CUPRAC assay (Figure 3c) proved to be a nonspecific method, providing a high but generalized antioxidant value (*p* > 0.05 for all compounds). The CUPRAC assay involves the reduction of copper ions, which suggests that the methyl substitution does not affect reducing power in this context.

The DPPH experiment (Figure 3a) revealed that the SB3 and SB4 compounds had excellent antioxidant activity (*p* > 0.05), but SB2 had much lower activity than the basic structure (SB1) and, the SB3 and SB4 compounds (*p* < 0.0001). Thus, a substituent at the ortho position may introduce steric effects that prevent interactions with free radicals. The same effect was observed using the TEAC method (Figure 3d), but significant differences were also noted between the SB1, SB3, and SB4 compounds. The trend of the antioxidant effect through this method was SB1 > SB3 > SB4 > SB2. The compound with the basic structure (SB1) without methyl was the most active, which suggests that the unsubstituted phenolic nucleus helps stabilize the radical species.

The antioxidant activity measured by the FRAP method (Figure 3b) highlighted that the SB1 and SB2 compounds have similar antioxidant activities (*p* > 0.05), while the SB3 and SB4 compounds exhibit a very low antioxidant activity (*p* > 0.05), suggesting that the methyl group in the meta and para positions affects reducing capacity, and the acidic environment might enhance this difference by altering the stability of the protonated forms [28,29].

The differences between compounds could be explained by the influence of methyl substituents on the phenolic ring, affecting their ability to donate electrons or hydrogen atoms (DPPH, TEAC), their reduction capacity in an acidic medium (FRAP), and the complexation of copper ions (CUPRAC). Modifying the location of CH3- groups can change a compound’s physical characteristics, such as water solubility, hydrophobic interactions, participation in van der Waals interactions, and logP [28,30]. These modifications might affect their capacity to donate electrons and stabilize the resultant radical species. Changes in CH_3_ group positions could affect a compound’s physico-chemical characteristics, bioavailability, and antioxidant activity. For example, solubility affects absorption in the gastrointestinal tract, while hydrophobic and van der Waals interactions affect the interactions with cell membranes or the proteins involved in an antioxidant response [31]. The placement of the CH3 group on the benzene ring has an impact on antioxidant potential, as it could affect antioxidant enzymes and how they interact with free radicals [32]. These characteristics can influence the behavior of the compound in the body, including its antioxidant activity, therapeutic suitability, tissue distribution, and bioavailability, all of which are directly related to changes in logP [33,34].

Through steric effects, methyl groups can modify antioxidant activity by changing how they interact with various target locations, such as antioxidant proteins or free radicals, as well as how stable and bioavailable they are in biological environments [35,36].

#### 2.2.2. Qualitative and Quantitative Evaluation of the Antimicrobial Activity of Thiourea Derivatives

Thiourea derivatives exhibit a wide range of biological activities, including antibacterial, anti-tuberculotic, antiparasitic, and antiarrhythmic effects. Their antimicrobial properties are associated with their ability to interfere with quorum sensing, an important mechanism by which microorganisms coordinate their collective behaviors. This phenomenon is thought to result from their structural similarity to N-acetyl-homoserine-lactone, an essential signaling molecule that plays an essential role in bacterial communication [37].

In the antimicrobial assays against *C. auris* strains, the SB2 compound, distinguished by an ortho-methyl substitution, exhibited a significantly stronger inhibitory effect compared to both the solvent control and the other tested compounds, as detailed in Table 3.

**Figure 4:**
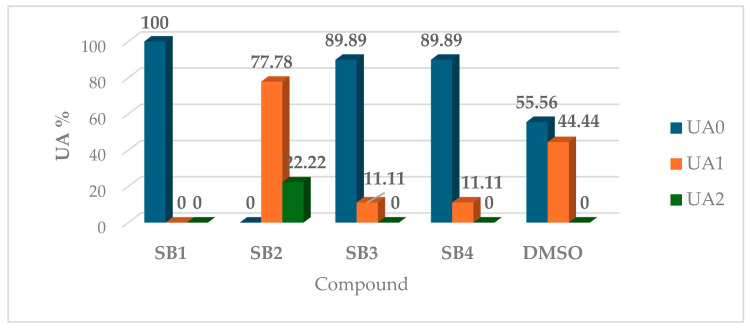
Distribution of arbitrary units determined by investigated compounds on *C. auris* strains.

The antifungal activity of the investigated compounds was assessed and classified based on their inhibitory potential, assigned as arbitrary units (UAs), as illustrated in Figure 4. Among the evaluated derivatives, SB2 demonstrated the highest efficacy, being the only compound to inhibit all tested strains, with inhibition rates ranging from 22.22% for UA2 to 77.78% for UA1. Compounds SB3 and SB4 displayed comparable antimicrobial activity, each achieving an inhibition rate of 11.11% against UA1. In contrast, SB1 exhibited no inhibitory effect against any of the nine tested strains, resulting in a UA0 classification across 100% of the cases.

Concerning the quantitative evaluation of antifungal activity performed by the microdilution method and the determination of the minimum inhibitory concentration (MIC) values of the thiourea derivatives on the analyzed *C. auris* strains, the results are included in Table 3.

According to Table 3, the inhibitory effect of the compounds depends both on their structure and on each individual strain. Thus, the average MIC values obtained ranged from 1.0417 to 5 mg/mL for SB1, 0.0781 to 0.625 mg/mL for SB2, 0.3125 to 5 mg/mL for SB3, and 0.625 to 1.6667 mg/mL for SB4.

The efficacy of the tested compounds significantly depended on the strain evaluated. SB2 showed the lowest MIC values and exhibited the highest antifungal activity. In contrast, SB3 had the highest MIC values for the majority of the strains and showed less efficacy than the other compounds. The antifungal activity of SB1 and SB4 varied depending on the examined strain, demonstrating an intermediate effect. A comparative analysis of compound activity between the strains revealed a possible differential resistance profile. The inhibitory mechanism of these thiourea derivatives is most likely based on their ability to disrupt fungal cell wall biosynthesis and biofilm formation through hydrogen bonding and electrostatic interactions with essential fungal enzymes, facilitated by the thiourea functional group and enhanced by ortho-methyl substitutions which increase molecular rigidity and electron density [38].

The *C. auris* DSM 21092 strain was highly sensitive to SB2 (MIC = 0.0781 mg/mL), while SB1 and SB4 showed MIC values above 1 mg/mL, indicating a lower efficacy. Also, strain 2851 responded well to SB2, but SB4 had a slightly increased MIC value (1.4583 mg/mL), suggesting variability in the sensitivity of this strain. Strains 6328 and 6816 were also susceptible to SB2, while for SB3, the MIC value was extremely high for strain 6328 (3.3333 mg/mL), confirming a possible intrinsic resistance to this compound. Similarly, for strain 9069, SB3 had the highest MIC value among all the strains tested (3.3783 mg/mL), indicating a strong resistance. Still, SB2 remained the most effective compound against this strain and also against strain 1370 (MIC = 0.3125 mg/mL), while SB3 maintained a high value (1.6667 mg/mL), but one that was slightly lower than that for strain 9069.

A consistent and reproducible action of SB2 is suggested by the correlation of MIC values with the standard deviations. For most strains, the standard deviations are small. In contrast, SB3 demonstrated significant variability in MIC values, especially for strains 6328 and 9069, suggesting an inconsistent antifungal efficacy.

SB2 is the most promising antifungal compound among those tested, showing high efficacy and good reproducibility, while SB3 appears to be the least effective.

The SB1–SB4 compounds can have their electron distribution and steric properties changed by adding primary aromatic amines with methyl radicals at different positions of the aromatic nucleus. This, in turn, changes how the molecules interact with biological targets. SB1 is the reference compound with no additional substituents on the benzene ring from aniline [39].

The superior efficacy of the SB2 compound can be explained by a detailed analysis of the structural, electrostatic, and steric influences of the substituents in their interaction with biological targets. This advantage is due to the fact that these compounds originate from the thiourea of 2-thiophene carboxylic acid [40].

The basic part of these substances is the 2-thiophenecarboxylic acid, which is a heterocycle with a thiophenic nucleus. Its aromatic and heteroatomic properties make it stable chemically and allow it to interact with other substances. The incorporation of a thiourea unit in its structure makes the groups more capable of forming hydrogen bonds and electrostatic interactions with the fungal enzymes involved in the growth of *C. auris* [41,42,43].

In SB2, introducing a methyl radical in the ortho position of the aromatic nucleus changes the electron density distribution. This change has an impact on the steric properties of the molecule as well as the ability of the molecule to interact with target proteins. Methyl radicals have an inductive effect (+I) that increases the electron density of the aromatic nucleus. This favors an electrostatic interaction with the enzymes involved in *C. auris* cell wall biosynthesis [44]. In addition, the ortho position of the methyl groups makes the molecule more rigid through internal steric interactions. This process reduces the probability of metabolic degradation of the molecule and increases the stability of the compound in a biological environment [45].

Comparison with the other compounds (SB1, SB3, and SB4) emphasizes the influence of substituent positioning on biological activity. The electronic spread changes in SB3 (meta substitution) and SB4 (para substitution). This might affect how well the molecules fit with fungal proteins in terms of sterics and electrostatics. SB3, having the methyl substituent in the meta position, shows an electronic distribution less favorable for direct interactions with the active sites, which is reflected in a higher MIC for most of the analyzed strains. SB4, with the methyl substituent in the para position, may have a polarizing effect that affects the electrostatic stability of the molecule, thus reducing antifungal efficacy [46,47,48].

Fungal adherence to surfaces is the first step in biofilm formation, which prolongs infections and causes antifungal resistant *C. auris*, an emerging pathogen with enhanced adherence and biofilm production.

**Figure 5:**
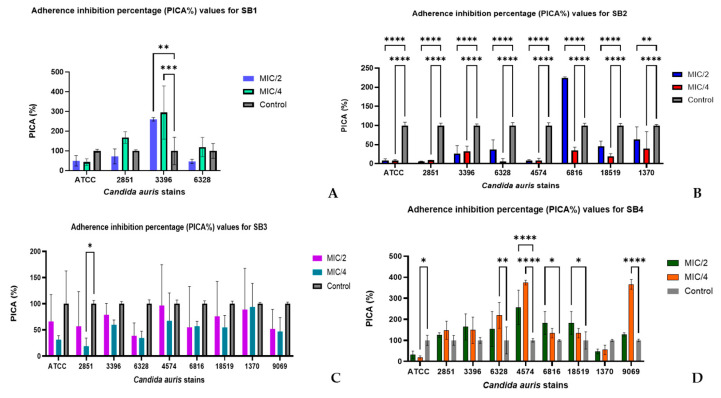
Adherence inhibition percentage (PICA%) values for SB1, SB2, SB3, and SB4 compared to the *C. auris* strains: (**A**) PICA% values for SB1 showing the highest inhibition against strain 3396 at both MIC/2 and MIC/4 concentrations; (**B**) PICA% values for SB2 revealing strong inhibition activity at MIC/2 and MIC/4 across most strains, particularly against 4574 and 6816; (**C**) PICA% values for SB3 indicating moderate and uniform adherence inhibition across all strains tested, with significant effects against DSM and 2851; (**D**) PICA% values for SB4 highlighting potent inhibition for several strains, especially 9069, 6816, and 4574, with statistically significant differences compared to the control. (* *p* < 0.05, ** p < 0.001, *** *p* < 0.001, **** p < 0.0001) (Dunnett’s multiple comparisons test).

The results in Figure 5 show how the SB1–SB4 compounds influence strain adherence to an inert substratum [49].

Among the compounds that were tested, SB2 caused a strong and consistent inhibition of adherence to the *C. auris* stains, as shown by the low values of the adherence inhibition percentage (PICA%). For example, for the reference *C. auris* DSM 21092, the PICA% was 7.9 ± 5.1 at MIC/2 and 7.87 ± 3.12 at MIC/4, indicating an almost complete reduction of the adherence compared to the control (100 ± 8.66).

The clinical strains also showed a similar pattern. SB2 significantly reduced adherence for strain 2851 (5.5 ± 1.35 at MIC/2 and 9.05 ± 0.36 at MIC/4), while the values for 4574 were comparable (7.88 ± 2.85 at MIC/2 and 8.49 ± 5.65 at MIC/41). These results indicate that SB2 functions effectively disrupt key molecular interactions involved in cell adherence, thus preventing the formation of a stable biofilm.

In contrast to SB2, which showed uniform efficacy, SB4 displayed selective activity, demonstrating high effectiveness against certain strains while being less active against others. The most pronounced effect was observed for the strain encoded 4574, where the PICA% was 256.7 ± 81.5 at MIC/2 and 374.6 ± 11 at MIC/4, indicating a significant reduction in adherence. In strain 2851, the PICA% was significantly higher (125.7 ± 11.3 for MIC/2 and 148.2 ± 42.6 for MIC/4), suggesting a lower susceptibility of this strain to SB4. A similar pattern was observed for strain 3396, where SB4 had a reduced efficiency (PICA% of 164.0 ± 61.9 for MIC/2 and 148.4 ± 62.1 for MIC/4).

In contrast, SB3 had variable results between strains and showed no obvious inhibition of adherence capacity. Although MIC/4 had a significant effect on strain 2851 (PICA% 18.98 ± 15.5), for most of the other strains, the values were high and close to those of the control. SB3 is not effective at inhibiting fungal adherence and cannot be considered a promising antifungal agent. With high PICA% values and high inter-replicate variability, SB1 was the least effective of all the compounds examined. For this compound, data on its effect on the four strains were considered, for the others the PICA% value greatly exceeded the control mean. For *C. auris* DSM 21092, SB1 failed to significantly reduce adherence, with a PICA% of 49.7 ± 25.9 for MIC/2 and 43.3 ± 16.8 for MIC/4. Strain 2851 showed a paradoxical increase in adherence to MIC/4 (PICA% 167.9 ± 29.3), suggesting high experimental variability or a possible adaptation of the strain to the compound.

The results indicate a strong correlation between the chemical structure of the SB1-SB4 compounds and their efficacy in inhibiting *C. auris* adherence. Of these, SB2 showed the highest inhibitory potential, probably due to the presence of the methyl substituent in the ortho position, which facilitates conformational stabilization and enhances electrostatic interactions with fungal cell surface components. Further studies are needed to further explore its mechanism of action as an antifungal agent. In contrast, SB3 and SB4, which possess methyl substituents in the meta and para positions, showed weaker and more selective activity. This is consistent with previous studies showing that meta and para substitutions can change the hydrophobicity and molecular flexibility of thiourea derivatives, therefore producing a different bioavailability and target affinity in fungal cells [50]. This indicates that the electron density distribution within the molecule significantly affects its ability to inhibit the adherence capacity. In contrast, SB1, lacking substituents on the aromatic core, showed the lowest efficiency except for one strain (3396), thus affirming the importance of structural modifications in modulating antifungal activity. It is important to note that in certain cases, particularly for SB1, paradoxical increases in the PICA% values were observed, likely due to strain-specific variability or adaptive responses at subinhibitory concentrations. Additionally, the high standard deviations observed for some data points in Figure 5 reflect biological variability between the replicates, emphasizing the need for further investigations on a larger number of strains.

The resistance of *C. auris* strains to the compounds can be explained by both biological mechanisms and chemical variables, in line with the results mentioned above. The capacity of *C. auris* to develop resistance against azoles and polyenes is well documented, primarily through *ERG* gene mutations and efflux pump overexpression [51,52]. Given the limited number of studies on the biofilm formation of *C. auris* under the influence of thiourea derivatives, the present study aims to evaluate the therapeutic potential of these substances in the treatment of nosocomial infections caused by *C. auris*.

The heterogeneity of *C. auris* strains isolated from a clinical hospital in Bucharest was demonstrated by indirect identification methods based on chromogenic culture media in combination with particular taxonomic techniques. The enzyme profile of these strains revealed the presence of hemolysins and esculin hydrolyzing enzymes, implying a significant pathogenic potential. Esculin, a coumarin derivative that exhibits antibacterial characteristics and efficacy against *C. albicans*, can be hydrolyzed by *C. auris* strains, potentially contributing to their pathogenesis. Although the enzymatic processes underlying this reaction remain poorly defined, new research indicates that esculin hydrolysis may serve as a unique virulence factor for invasive *C. auris* strains, necessitating further investigation into its clinical significance [52].

Recent studies have demonstrated the significant resistance of *C. auris* to fluconazole and amphotericin B, a trend that aligns with our findings [22]. These results emphasize the need to develop improved diagnostic strategies and targeted antifungal treatments [22,53,54,55,56].

A study conducted in a clinical hospital in Bucharest demonstrated the significant resistance of *C. auris* to fluconazole after analyzing samples from 13 patients. However, a favorable therapeutic response was noted when it was tested with amphotericin B, micafungin, and caspofungin [51]. The increasing prevalence of multidrug-resistant (MDR) *C. auris* strains is recognized as a significant global health threat. A recent genomic surveillance study found a strong link between *C. auris* being resistant to fluconazole and changes in the *ERG* gene, especially in clade III isolates, which have been linked to large-scale hospital outbreaks. The rapid evolution and dispersal of fluconazole-resistant strains suggest that selective pressures, probably driven by widespread antifungal use, have contributed to the persistence of resistant *C. auris* populations [57,58].

Given the clinical context, this resistance to classical antifungals requires the discovery of new molecules with antifungal actions. Starting from the idea of obtaining new compounds simply and cheaply, four new substances with potential biological activity were tested in this study.

Bădiceanu et al. (2018) have characterized the compounds in the literature as exhibiting antibacterial properties [53,54]. The compounds showed efficacy against *C. albicans*. The antifungal efficacy of methylated derivatives at the ortho, meta, and para positions on the benzene nucleus was evaluated against *C. auris* strains that come from a nosocomial environment. The results from the quantitative and qualitative evaluation of the antifungal impact indicated the enhanced efficacy of the ortho derivative relative to the other derivatives [59]. The impact of the position and nature of the radicals on the aromatic nucleus regarding the biological effect of a compound has been established. A methyl radical is stable but is not as reactive as a hydroxyl radical, a methoxy radical, or halogenated radicals (-CI3, -CBr3, -CCl3, etc.) [12].

Various compounds have been evaluated for their ability to inhibit *C. auris* biofilm formation. Among these compounds, antifungal agents such as echinocandins (caspofungin, micafungin, and anidulafungin) showed marked efficacy when compared with azoles. Antifungal peptides and small molecules such as histatin-5 and farnesol disrupt cell adhesion and quorum sensing, preventing biofilm formation and growth [10,59]. Natural compounds have been investigated for their potential to inhibit biofilm formation. Essential oils like tea tree and eucalyptus oils, as well as plant extracts like garlic and curcumin, have been shown to reduce biofilm biomass and break up the extracellular matrix. These natural substances frequently operate through various mechanisms, such as disrupting cell membranes and interfering with the signaling pathways that are crucial for biofilm development [60,61,62,63].

To increase the ability to inhibit the biofilm formation, some researchers have proposed integrating this type of thiourea into metal complexes. An increased antifungal effect has been demonstrated in cases employing the use of the nickel and copper complexes of some thiourea derivatives, proving an in vitro antimicrobial effect on *C. albicans*, *C. krusei*, *C. glabrata*, *C. tropicalis*, and *C. parapsilosis* strains [64].

Bădiceanu et al. (2014) demonstrated the influence of these structural variations on antibacterial effectiveness [14]. Numerous thiourea derivatives were synthesized by altering their fundamental structure with diverse aromatic and aliphatic substituents. These modifications influence the compound’s capacity to interact with microbial cell membranes and intracellular targets [14]. The inclusion of electron-donating groups, such as methoxy or hydroxyl groups on the aromatic ring, typically enhances antibacterial action. These groups can enhance the electron density on a thiourea compound, promoting stronger interactions with microbial enzymes and interfering with essential cellular activities. Electron-withdrawing groups, such as nitro (-NO_2_) or halogen (-X) atoms, might diminish antibacterial activity, but they can improve the precision for particular pathogenic targets [64]. Thiourea derivatives can get through microbial cell walls and membranes differently depending on how flexible and flat they are. Other than that, substituents like the polar groups that make chemicals more soluble in biological media can also make thioureas more bioavailable and effective overall.

Bădiceanu et al. (2009) revealed synergistic interactions between thioureas and conventional antibiotics, indicating that structural modifications targeted at enhancing interactions with microbial targets may augment antifungal efficacy [12].

The antibacterial efficacy of thioureas synthesized from 2-thiophenecarboxylic acid is significantly influenced by their structural characteristics, indicating their potential for the development of treatments against resistant pathogens such as *C. auris*. Considering the epidemiological significance and pathogenicity of *C. auris*, the development of new antifungal drugs is imperative.

#### 2.2.3. Hemocompatibility

**Figure 6:**
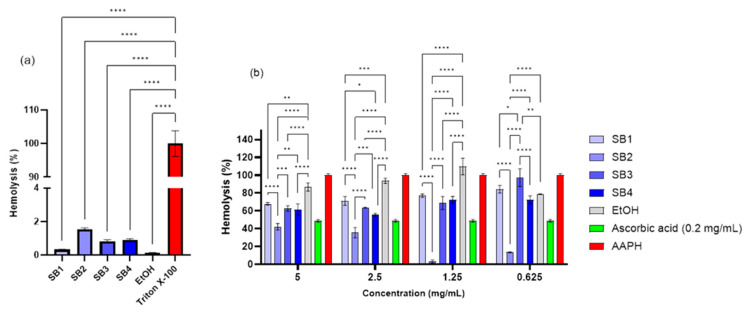
Anti-hemolytic activity of newly synthesized compounds: (**a**) hemolysis (%) induced by SB1, SB2, SB3, SB4, and ethanol 70% relating to Trypton X-100 and (**b**) antihemolytic activity of these compounds against the AAPH-induced oxidative hemolysis of ram erythrocytes (* *p* < 0.05, ** *p* < 0.01, *** *p* < 0.001, **** *p* < 0.0001).

To analyze anti-hemolytic impact based on an erythrocyte membrane lipid peroxidation mechanism, the hemolytic effect was examined to determine that compounds SB1, SB2, SB3, and SB4 display a very low hemolysis, less than 5%, indicating little cytotoxicity towards erythrocytes. The solvent used, 70% EtOH, was evaluated under the same conditions as the samples and was found to not induce considerable hemolysis, as predicted, because it is not a powerful hemolytic agent, and the hemolysis induced by the samples does not appear to be caused by the solvent used. As a positive hemolysis control, 1% Triton X-100 was used, against which all compounds were shown to have significantly reduced hemolysis (*p* < 0.0001). According to Figure 6a, hemolysis increased in the following order: EtOH < SB1 < SB3 < SB4 < SB2, but the differences between the hemolysis induced by each compound were not statistically significant (*p* > 0.05).

The hemolysis effects of compounds SB1–SB4 vary, with SB2 having the greatest impact. These discrepancies can be related to structural changes, such as the methyl group’s position in ortho on the aromatic ring. These changes influence the compound’s stability and interactions with erythrocyte cell membranes [65]. For example, SB2’s methyl group placement may improve its interaction with membrane components, resulting in increased hemolysis.

All samples were determined to be non-hemolytic; hence, their ability to protect against AAPH-induced hemolysis was further investigated. AAPH was thought to cause 100% hemolysis since it is a pro-oxidant molecule that causes oxidative stress. The chemical with the basic structure (SB1) had the poorest anti-hemolytic effect; Figure 6b shows that hemolysis rises as concentration decreases. The SB2 compound had the greatest hemolytic index (1.54 ± 0.09%), but it also had the strongest anti-hemolytic effect. However, this effect decreases with concentration, indicating a pro-oxidant action at higher levels. Higher concentrations of SB2 may have a pro-oxidant effect by interacting with the cellular environment to form reactive oxygen species (ROS), a mechanism already observed for other compounds with antioxidant properties [66,67]. This property may affect its antifungal efficiency, since it may cause fungal cells to experience intracellular oxidative stress [68]. So, one potential mechanism of action for antifungal activity might be this pro-oxidant effect. The SB3 and SB4 compounds follow a similar trend to SB1, with no statistically significant changes (*p* > 0.05). Ascorbic acid was utilized as a benchmark for the anti-hemolytic activity, which confirmed its antioxidant properties [69]. All compounds showed a dose-dependent trend, with substances SB1–SB4 considerably lowering AAPH-induced hemolysis, indicating an anti-hemolytic ability and maybe an antioxidant impact. The location of the methyl group affects antioxidant activity, with SB2 (ortho) having a much higher anti-hemolytic action than SB1 (without methyl), SB3 (meta), and SB4 (para).

## 3. Materials and Methods

### 3.1. Strains Identification

A total of eight *C. auris* strains isolated from hospitalized patients at the National Institute of Pneumophthiology Marius Nasta, in Bucharest, Romania, from January to May 2024 were collected. The strains were isolated using a Sabouraud Dextrose Agar (SDA) culture medium (Alliance Bio Expertise, Bruz, Brittany, France) from different isolation sources (Table 4) and identified using a CHROMagar™ Candida Plus medium (Chromagar, Saint-Denis, France), a chromogenic medium used for the direct identification of *Candida* species [60], as well as a matrix-assisted laser desorption/ionization time-of-flight [MALDI-TOF MS MBT Smart, with MSP 96 target polished steel BC (Bruker system, Berlin, Germany)] technique. In addition, one control laboratory strain *C. auris* DSM 21092 was selected as a positive control because its virulence factors have been well characterized. The isolates were stored in glycerol stock at −20 °C until required. All strains were collected with an approval by the human Research Ethics Committee of the National Institute of Pneumophthiology Marius Nasta (Approval Number: 12255/17 June 2024), in accordance with the ethical guidelines outlined in the Declaration of Helsinki.

### 3.2. Determination of Antifungal Susceptibility Profiles

Antifungal susceptibility testing was performed using the E-test method, a quantitative technique that determines the MIC values of antifungal agents against fungal pathogens. For this, 1 McFarland standardized suspension obtained from the tested strains and placed into sterile water was inoculated onto a SDA culture media, followed by the placement of E-test strips and incubation at 37 °C for 48 h. Susceptibility testing was conducted for fluconazole (FL, 0.016–256 µg/mL), micafungin (MYC, 0.002–32 µg/mL), amphotericin B (AMB, 0.002–32 µg/mL), caspofungin (CAS, 0.002–32 µg/mL), and flucytosine (FC, 0.002–32 µg/mL). MIC values were interpreted according to the 2018 Clinical and Laboratory Standards Institute (CLSI) M60 guidelines [6,61].

### 3.3. Determination of Soluble Virulence Factors Production

The production of soluble virulence factors by *C. auris* strains, specifically metabolic enzymes (amylase, hemolysins, aesculin hydrolysis, caseinases, and lecithinase), was assessed using the established solid culture media protocols. For hemolysin activity, *C. auris* isolates were cultured on an SDA supplemented with 5% sheep blood, where hemolysin production was identified by the appearance of a clear, colorless halo surrounding the colonies after 24 h of incubation at 37 °C. Egg yolk agar (2.5%), which was used to test lecithinase activity, showed positive results when a clear zone formed around the inoculum after 24 h of incubation at 37 °C. Caseinase activity was tested on an agar that had 15% soluble casein. After 24 h at 37 °C, the presence of a white precipitate around the colonies showed that the enzyme was active. Amylase production was determined using a starch agar (1%), where, upon the addition of Lugol’s iodine solution (Sigma-Aldrich, St. Louis, MO, USA), a yellow halo around the colonies signified starch hydrolysis, contrasting with the blue coloration of the remaining medium. Aesculin hydrolysis was tested on an agar supplemented with Fe^3+^ citrate. Positive activity was indicated by a black precipitate forming around the colonies after 24 h of incubation at 37 °C [62,63]. This method offers a thorough evaluation of the enzymatic virulence factors linked to *C. auris*, enhancing the understanding of its pathogenic mechanisms.

### 3.4. Biological Properties of New Synthetic Compounds

#### Physico-Chemical Characterization of the Thiourea Derivatives

**Figure 7:**
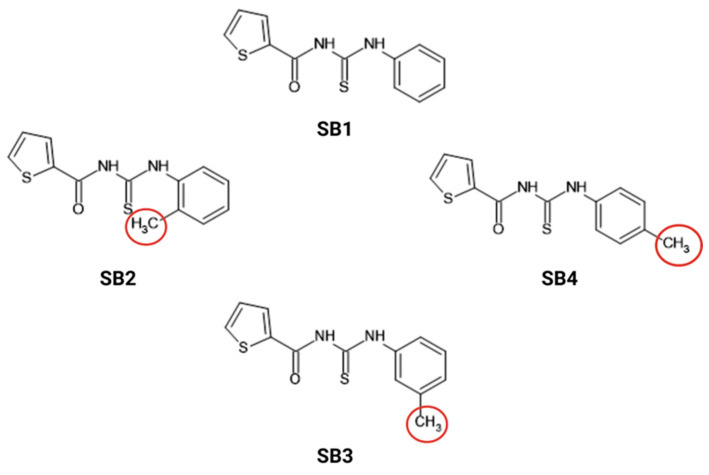
The chemical structure of the compounds

In the Laboratory of Pharmaceutical Chemistry of the Faculty of Pharmacy of the University of Medicine and Pharmacy “Carol Davila” in Bucharest, four thiourea derivatives were obtained following the reaction of 2-thionyl-isothiocyanate with various aromatic primary amines. The 2-thionyl-isothiocyanate was obtained from the corresponding acid chloride with ammonium thiocyanate in dry acetone under reflux. The necessary 2-thiophenecarboxilic acid chloride was obtained from 2-thiophenecarboxilic acid and thionyl chloride. The compounds were characterized by their physical–chemical properties and their structure was confirmed by ^1^H-NMR and ^13^C-NMR spectra, according to Bădiceanu, 2009 [12]. The structure of the compounds is described in Figure 7 [12].

The compounds are yellowish-white powders with increased stability, without being photosensitive or thermolabile, and poorly hygroscopic. They are insoluble in water, soluble in organic solvents (dimethyl sulfoxide—DMSO, alcohols), and their characteristics are determined by their non-polar molecules [12].

### 3.5. Antioxidant Activity

The DPPH assay was carried out using the method reported by Madhu et al. [64], with a few adjustments. The reaction mixture consisted of 500 µL of a sample/standard and 500 µL of 0.3 mM a DPPH radical methanol solution. The absorbance was measured at λ = 517 nm after 25 min of dark incubation and centrifugation for 5 min at 10,000 rpm, for the precipitate separation. The Trolox calibration curve included values ranging from 5 to 100 µM (R^2^ = 0.9977). The results were expressed as µM Trolox equivalents per mg of compound.

The FRAP test was performed according to the protocol described by Multescu et al. [69] with a few adjustments. The reaction mixture consisted of 475 µL of a FRAP reagent and 25 µL of a sample/standard. The absorption was measured at λ = 593 nm after 25 min of incubation at 37 °C in the dark and 5 min of centrifugation at 10,000 rpm. Various doses (25–250 µM) were generated from a standard Trolox solution (1 mM), with a correlation coefficient of R^2^ = 0.9998. The results were expressed as µM Trolox equivalents per mg of compound.

The CUPRAC assay was performed using the procedure given by Bostiog et al. [70]. Specifically, 120 μL of sample/standard solutions of different concentrations were combined with 100 μL of CuCl_2_ (10 mM) and 100 μL of neocuproine (7.5 mM). After 25 min of incubation and centrifugation at 10,000 rpm for 5 min, absorbance was measured at 450 nm. The Trolox calibration curve used concentrations ranging from 0.100 to 1.5 mM (R^2^ = 0.9996). The results were expressed as µM Trolox equivalents per mg of compound.

The Trolox Equivalent Antioxidant Capacity (TEAC) test was carried out as described by Multescu et al. [69] with minor modifications. A stock solution of ABTS^+^ was created by combining a 7 mM ABTS solution with 2.45 mM of potassium persulphate. Before being used, the mixture was kept in the dark for 12–16 h to allow for the complete formation and stabilization of the cationic radical ABTS^+^. Diluting ABTS^+^ with methanol yielded a workable solution with an absorbance of about 0.70. The reaction mixture included 100 μL of a sample/standard and 900 μL of a working ABTS^+^ solution. It was incubated for 25 min in the dark and centrifuged for 5 min at 10,000 rpm. The standard curve was linear from 20 to 150 µM Trolox (R^2^ = 0.9953). The results were given as µM Trolox per mg of substance.

### 3.6. Antimicrobial Activity

#### 3.6.1. Qualitative Testing of the Antifungal Activity of the Test Compounds on *C. auris* Strains

A qualitative assessment of the antifungal activity of the test compounds was conducted using a modified disk diffusion assay, based on the Clinical and Laboratory Standards Institute (CLSI) M44 standard (2018) [71,72]. Stock solutions were prepared at a concentration of 10 mg/mL in dimethyl sulfoxide (DMSO). Standardized fungal suspensions, adjusted to a 1.0 McFarland standard in sterile water, were obtained from *Candida* strains, including the reference strain *Candida auris* DSM 21092, and cultured on an SDA. Subsequently, each suspension was evenly spread onto the surface of SDA plates, followed by the application of 10 µL of each investigated solution. DMSO served as the solvent control. The plates were then incubated at 37 °C for 24 h, after which the diameters of the growth inhibition zones were measured. The results were categorized into arbitrary inhibition units (UAs): 0 (no inhibition), 1 (10–20 mm inhibition zone), and 2 (≥21 mm inhibition zone) [73].

#### 3.6.2. Quantitative Evaluation of the Antifungal Activity of Test Compounds on *C. auris* Strains—The MIC Determination

The quantitative evaluation of antifungal efficiency was performed using the serial binary microdilution method in 96-well plates (ranged between 5 and 0.009 mg/mL), in an RPMI 1640 broth medium (American Biorganics, Buffalo, NY, USA) to which a standardized suspension corresponding to 1 McFarland obtained from the tested strains in sterile water was added to a final concentration of 10%, in a total volume of 100 µL. After 24 h at 37 °C, the results were analyzed by determining the optical density at 620 nm using a Thermo Scientific™ Multiskan™ GO Microplate Spectrophotometer (17) (Thermo Fisher Scientific, Waltham, MA, USA). The lowest concentration of the investigated solution that completely inhibited the strains’ growth was established as the minimum inhibitory concentration (MIC), based on the results analyzed in triplicate and the blank subtraction.

#### 3.6.3. Evaluation of the Influence of the Tested Substances on Microbial Adherence Capacity to the Inert Substratum

The effect of the investigated solutions on the microbial adherence to an inert substratum was assessed using the crystal violet microtitration method. For this purpose, *Candida* strains were cultured in the presence of subinhibitory concentrations of the solutions (MIC/2 and MIC/4) following the protocol for a quantitative antimicrobial assessment. After incubation for 24 h at 37 °C, adherent bacterial cells were fixed with 99% methanol, stained with 1% crystal violet, and resuspended in 33% acetic acid. The percentage of adherence inhibition (%PICA) was determined using the following formula: %PICA = (As − Ablank) × 100/(Ac − Ablank), where As is the absorbance at 490 nm of the treated samples, and Ac is the absorbance at 490 nm of the control as previously described in [74].

### 3.7. Hemocompatibility Assays

#### 3.7.1. Hemolytic Index

The hemolysis test was carried out using ram red blood cells (RBCs). To avoid blood clots, 9 mL of a blood sample was combined with 1 mL of 10% citric acid dextrose (due to its mechanism of action on calcium ions, citric acid acts as a chelating agent while dextrose helps maintain the viability of blood cells). The tube was then centrifuged at 5000 rpm for 10 min. The surface containing platelet-poor plasma was removed, and the sediment containing red blood cells was resuspended in 10 mL of phosphate-buffered saline (0.1 M, pH 7.4). Finally, the cells were suspended in PBS. Marinas et al. [75] detailed the procedure, with some adjustments. The 250 μL of erythrocyte suspension was combined with 250 μL of several concentrations of newly synthesized compound solutions adjusted with 0.9% NaCl, comparing the results with those obtained in the antifungal activity tests. The tubes were then gently inverted and incubated at 37 °C for 60 min. Positive and negative controls were made by adding the same quantity of erythrocyte suspension to 250 μL of 1% Triton-X100 and PBS, respectively. After incubation, the samples were centrifuged at 5000 rpm for 10 min, and the resulting supernatant was carefully divided into 96-well plates. The absorbance of the supernatant was measured at 540 nm, the maximum absorption of free hemoglobin in a solution.

#### 3.7.2. Antihemolytic Activity

The anti-hemolytic capability of the newly synthesized compounds was determined using a previously established spectrophotometric method [76], with minor modifications. A stock solution of 10 mg/mL was prepared for each newly synthesized compound in 70% ethanol (the working concentrations were 5–0.1625 mg/mL in PBS, pH = 7.4). A total of 100 µL of the sample was mixed with 400 µL of a 10% erythrocyte suspension. After 20 min of incubation at 37 °C, 200 µL of AAPH (2, 2′-Azobis (2-amidinopropane) dihydrochloride) (final concentration was 200 mM) was added, inducing a significant but controllable level of hemolysis to effectively evaluate the effects of the tested compound. The mixes were incubated for 4 h at 37 °C, to ensure sufficiently advanced oxidation, thus allowing a clear evaluation of the protective capacity of the tested compound against AAPH-induced oxidative stress. The mixes were incubated for 4 h at 37 °C. The samples were then centrifuged at 5000 rpm for 10 min, and the absorbance of the supernatant was measured at 540 nm. The relative hemolysis was calculated as compared to the hemolysis in the negative control treated with AAPH, which was defined as total hemolysis at 100%. The positive control was PBS that had not been treated with AAPH. Each set of studies was carried out in triplicate, and inhibitory activity was represented as a percentage of hemolysis inhibition. A stock solution of ascorbic acid (0.2 mg/mL) as an antioxidant control was handled using the same procedure as with the samples.

### 3.8. Statistical Analysis

All experiments were conducted in triplicate to ensure reproducibility. A statistical analysis was performed using GraphPad Prism (version 10). To evaluate the impact of the four tested thiourea derivatives on the adherence capacity of C. auris strains to an inert substrate, a one-way analysis of variance (ANOVA) was applied. Subsequently, Dunnett’s multiple comparison test was used to compare the treated groups with the untreated control groups. For the evaluation of antioxidant and hemolytic activity, one-way ANOVA was used employing Tukey’s method for multiple comparisons, with a single pooled variance. A *p*-value < 0.05 was considered statistically significant.

## 4. Conclusions

This study addresses the limited research on *C. auris* virulence and antifungal resistance in Romanian hospitals by assessing phenotypic variability, virulence factors, and treatment options. It confirms *C. auris*’s resistance to fluconazole and highlights the potential of four thiourea derivatives (SB1–SB4), with SB2 (ortho-methylated) showing the strongest antifungal and anti-adherence effects. The position of the methyl group significantly affects antioxidant activity and hemolytic protection, with SB2 also demonstrating strong antioxidant and anti-hemolytic properties. These findings suggest a promising therapeutic potential and warrant further clinical investigation.

## Figures and Tables

**Table 1:** E-test results on antifungal susceptibility of *C. auris* strains.

Strain Code	Antifungal—MIC Value (µg/mL): Interpretation According to CLSI Standard									
	Fluconazole		Micafungin		Flucytosine		Caspofungin		Amphotericin B	
1370	>256	R	0.047	S	0.094	S	0.38	S	>32	R
4574	>256	R	0.023	S	0.38	S	0.75	S	>32	R
18519	>256	R	0.032	S	0.94	S	0.75	S	>32	R
3396	>256	R	0.047	S	1.50	S	1.5	S	3	R
9096	>256	R	0.125	S	0.50	S	0.75	S	>32	R
6816	>256	R	0.064	S	0.50	S	0.50	S	>32	R
6328	>256	R	2	S	0.50	S	>32	R	>32	R
2851	128	R	0.023	S	0.38	S	1	S	>32	R

Legend: R—resistant; S—sensible.

**Table 2:** Soluble virulence factors identified in *C. auris.*

Strain Code	Hemolysins	Lecithinase	Amylase	Aesculin Hydrolysis	Caseinase
1370	+++	-	-	++	-
4574	+++	-	-	++	-
18519	+++	-	-	++	-
3396	+++	-	-	+++	-
9096	+++	-	-	++	-
6816	+++	-	-	++	-
6328	+++	-	-	++	-
2851	+++	-	-	++	-

Legend: The level of soluble virulence factor expression: - without enzymatic activity; ++: moderate activity; +++: strong enzymatic activity (large halo).

**Table 3:** Antifungal activity of thiourea derivatives on selected *C. auris* strains.

Strain	Compound				
	SB1	SB2	SB3	SB4	DMSO
*C. auris* DSM 21092	1.0417	0.0781	0.63	1.35	2.50
6816	0.0781	0.625	1.35	0.94	1.25
6328	1.0417	0.1563	3.33	1.04	0.63
4574	1.25	0.3646	5.00	0.63	1.25
3396	0.3646	2.0833	1.67	1.25	10.00
2851	1.3542	0.2865	0.31	1.46	5.00
18519	5	0.2083	2.08	1.67	10.00
1370	5	0.3125	1.67	0.83	2.50
9069	1.875	0.5208	3.38	1.25	10.00

Legend: MIC—Minimum inhibitory concentration (mg/mL).

**Table 4:** Isolation sources of nosocomial *C. auris* strains.

Strain Code	Isolation Source
1370	Bronchial aspirate
2851	Perianal swab
3396	Bronchial aspirate
4574	Vaginal discharge
6328	Eschar
6816	Urine
9069	Urine
18519	Urine

## Data Availability

The original contributions presented in this study are included in the article. Further inquiries can be directed to the corresponding authors.

## References

[1] Borton D. (2024). *Candida auris*: Emerging fungal pathogen in the US. Nursing.

[2] Badiee P., Alborzi A. (2011). Susceptibility of clinical *Candida* species isolates to antifungal agents by E-test, Southern Iran: A five year study. Iran J. Microbiol..

[3] De Gaetano S., Midiri A., Mancuso G., Avola M.G., Biondo C. (2024). *Candida auris* Outbreaks: Current Status and Future Perspectives. Microorganisms.

[4] Cristina M.L., Spagnolo A.M., Sartini M., Carbone A., Oliva M., Schinca E., Boni S., Pontali E. (2023). An Overview on *Candida auris* in Healthcare Settings. J. Fungi.

[5] Kim J.S., Cha H., Bahn Y.S. (2024). Comprehensive Overview of *Candida auris*: An Emerging Multidrug-Resistant Fungal Pathogen. J. Microbiol. Biotechnol..

[6] Keighley C., Garnham K., Harch S.A.J., Robertson M., Chaw K., Teng J.C., Chen S.A. (2021). *Candida auris:* Diagnostic Challenges and Emerging Opportunities for the Clinical Microbiology Laboratory. Curr. Fungal Infect. Rep..

[7] Franco L.C., Ahmed M., Kendra C.G., Sperling R.M., Van Benten K., Lavik J., Emery C.L., Relich R.F., Gavina K. (2024). Validation of a qualitative real-time PCR assay for the detection of *Candida auris* in hospital inpatient screening. J. Clin. Microbiol..

[8] Chowdhary A., Jain K., Chauhan N. (2023). *Candida auris* Genetics and Emergence. Annu. Rev. Microbiol..

[9] Ilie M.I. (2023). *Candida auris*: The unwelcome superfungus. Farmacia.

[10] Alexander B.D. (2017). Performance Standards for Antifungal Susceptibility Testing of Yeasts.

[11] Ahmad S., Alfouzan W. (2021). *Candida auris*: Epidemiology, Diagnosis, Pathogenesis, Antifungal Susceptibility, and Infection Control Measures to Combat the Spread of Infections in Healthcare Facilities. Microorganisms.

[12] Badiceanu C.D., Missir A.V. (2009). Synthesis and characterization of some new thioureides of 2-thiophenecarboxylic acid with potential pharmacological activity. Rev. Roum. Chim..

[13] Chniti I., Thebti A., Sanhoury M.A.K., Cherif H.O., Chehidi I. (2020). Synthesis, In vitro Antibacterial and Antifungal Activities of Trifluoroalkyl-N, N’-Disubstituted Thioureas. Org. Med. Chem. Int. J..

[14] Daniela Badiceanu C., Draghici C., Missir A vasile Carmen Chifiriuc M., Elena Stecoza C. (2014). Synthesis, Characterization and Antimicrobial Evaluation of Some New Thioureas derived from 3-thiophenecarboxylic Acid. Rev. Chim..

[15] (2024). Prevention of Invasive Infections [Internet]. *Candida auris* (*C. auris*). https://www.cdc.gov/candida-auris/hcp/infection-control/invasive-infection-prevention.html.

[16] Hata D.J., Humphries R., Lockhart S.R. (2020). *Candida auris*: An Emerging Yeast Pathogen Posing Distinct Challenges for Laboratory Diagnostics, Treatment, and Infection Prevention. Arch. Pathol. Lab. Med..

[17] Rudramurthy S.M., Chakrabarti A., Paul R.A., Sood P., Kaur H., Capoor M.R., Kindo A.J., Marak R.S., Arora A., Sardana R. (2017). *Candida auris* candidaemia in Indian ICUs: Analysis of risk factors. J. Antimicrob. Chemother..

[18] Weinstein M.P. (2018). Performance Standards for Antimicrobial Susceptibility Testing.

[19] Chaabane F., Graf A., Jequier L., Coste A.T. (2019). Review on Antifungal Resistance Mechanisms in the Emerging Pathogen *Candida auris*. Front. Microbiol..

[20] Rybak J.M., Barker K.S., Muñoz J.F., Parker J.E., Ahmad S., Mokaddas E., Abdullah A., Elhagracy R.S., Kelly S.L., Cuomo C.A. (2022). In vivo emergence of high-level resistance during treatment reveals the first identified mechanism of amphotericin B resistance in *Candida auris*. Clin. Microbiol. Infect..

[21] Rybak J.M., Muñoz J.F., Barker K.S., Parker J.E., Esquivel B.D., Berkow E.L., Lockhart S.R., Gade L., Palmer G.E., White T.C. (2020). Mutations in TAC1B: A Novel Genetic Determinant of Clinical Fluconazole Resistance in *Candida auris*. mBio.

[22] Stanciu A.M., Florea D., Surleac M., Paraschiv S., Oțelea D., Tălăpan D., Popescu G.A. (2023). First report of *Candida auris* in Romania: Clinical and molecular aspects. Antimicrob. Resist. Infect. Control.

[23] Frías-De-León M.G., Hernández-Castro R., Vite-Garín T., Arenas R., Bonifaz A., Castañón-Olivares L., Acosta-Altamirano G., Martínez-Herrera E. (2020). Antifungal Resistance in *Candida auris*: Molecular Determinants. Antibiotics.

[24] (2024). Clinical Treatment of Fungal Diseases: Antifungals [Internet]. Fungal Diseases. https://www.cdc.gov/fungal/hcp/clinical-care/index.html.

[25] Jaiswal N., Kumar A. (2022). HPLC in the discovery of plant phenolics as antifungal molecules against *Candida* infection related biofilms. Microchem. J..

[26] Watkins R.R., Gowen R., Lionakis M., Ghannoum M. (2022). Update on the Pathogenesis, Virulence, and Treatment of *Candida auris*. Pathog. Immun..

[27] Balows A. (2003). Manual of clinical microbiology 8th edition. Diagn. Microbiol. Infect. Dis..

[28] Charlton N.C., Mastyugin M., Török B., Török M. (2023). Structural Features of Small Molecule Antioxidants and Strategic Modifications to Improve Potential Bioactivity. Molecules.

[29] Todorov L., Saso L., Kostova I. (2023). Antioxidant Activity of Coumarins and Their Metal Complexes. Pharmaceuticals.

[30] Pinheiro P.d.S.M., Franco L.S., Fraga C.A.M. (2023). The Magic Methyl and Its Tricks in Drug Discovery and Development. Pharmaceuticals.

[31] Ahmed T., Aljaeid B. (2016). Preparation, characterization, and potential application of chitosan, chitosan derivatives, and chitosan metal nanoparticles in pharmaceutical drug delivery. Drug Des. Dev. Ther..

[32] Huang X., Eggart D., Qin G., Sarma B.B., Gaur A., Yang J., Pan Y., Li M., Hao J., Yu H. (2023). Methyl radical chemistry in non-oxidative methane activation over metal single sites. Nat. Commun..

[33] Truzzi F., Tibaldi C., Zhang Y., Dinelli G., D’Amen E. (2021). An overview on dietary polyphenols and their Biopharmaceutical Classification System (BCS). Int. J. Mol. Sci..

[34] Cornea A.C., Marc G., Ionuț I., Moldovan C., Stana A., Oniga S.D., Pîrnău A., Vlase L., Oniga I., Oniga O. (2025). Synthesis, characterization, and antioxidant activity Evaluation of new N-Methyl substituted Thiazole-Derived polyphenolic compounds. Molecules.

[35] Zhang Y., Wu S., Qin Y., Liu J., Liu J., Wang Q., Ren F., Zhang H. (2017). Interaction of phenolic acids and their derivatives with human serum albumin: Structure–affinity relationships and effects on antioxidant activity. Food Chem..

[36] Burton G.W., Doba T., Gabe E., Hughes L., Lee F.L., Prasad L., Ingold K.U. (1985). Autoxidation of biological molecules. 4. Maximizing the antioxidant activity of phenols. J. Am. Chem. Soc..

[37] Delcaru C., Alexandru I., Podgoreanu P., Grosu M., Stavropoulos E., Chifiriuc M., Lazar V. (2016). Microbial Biofilms in Urinary Tract Infections and Prostatitis: Etiology, Pathogenicity, and Combating strategies. Pathogens.

[38] Pingaew R., Sinthupoom N., Mandi P., Prachayasittikul V., Cherdtrakulkiat R., Prachayasittikul S., Ruchirawat S., Prachayasittikul V. (2017). Synthesis, biological evaluation and in silico study of bis-thiourea derivatives as anticancer, antimalarial and antimicrobial agents. Med. Chem. Res..

[39] Liu S. (2014). Where does the electron go? The nature of ortho / para and meta group directing in electrophilic aromatic substitution. J. Chem. Phys..

[40] Gardarsson H., Schweizer W.B., Trapp N., Diederich F. (2014). Structures and Properties of Molecular Torsion Balances to Decipher the Nature of Substituent Effects on the Aromatic Edge-to-Face Interaction. Chem.–Eur. J..

[41] Crom W.R. (1992). Effect of chirality on pharmacokinetics and pharmacodynamics. Am. J. Health-Syst. Pharm..

[42] El-Zahed M.M., Kiwaan H.A., Farhat A.A.M., Moawed E.A., El-Sonbati M.A. (2023). Anticandidal action of polyurethane foam: A new modifier with functionalized isothiouronium group. Iran. Polym. J..

[43] Neacsu A., Badiceanu C., Stoicescu C., Chihaia V. (2024). DFT Studies on Physicochemical Properties and Spectral Data of 2-Thiophene Carboxylic Acid Thiourea Derivatives. Chem. Proc..

[44] Jensen H.H., Bols M. (2006). Stereoelectronic Substituent Effects. Acc. Chem. Res..

[45] Limban C., Marutescu L., Chifiriuc M.C. (2011). Synthesis, Spectroscopic Properties and Antipathogenic Activity of New Thiourea Derivatives. Molecules.

[46] Malavia-Jones D., Farrer R.A., Stappers M.H.T., Edmondson M.B., Borman A.M., Johnson E.M., Lipke P.N., Gow N.A. (2023). Strain and temperature dependent aggregation of *Candida auris* is attenuated by inhibition of surface amyloid proteins. Cell Surf..

[47] Roslan N., Bunnori N.M., Halim K.B.A., Kassim K., Aluwi M.F.F.M., Ngah N. (2022). Recent development on the synthesis of thiourea derivatives and effect of substituents on the anticancer activity: A short review. Malays. J. Anal. Sci..

[48] Kadhim M.M., Tomi I.H.R., Khadom A.A. (2023). Prediction of the corrosion inhibition efficiency and antibacterial activity of 1,2,4-oxadiazole derivatives using DFT and docking analysis: Effect of alkoxy chain length. J. Adhes. Sci. Technol..

[49] Toepfer S., Lackner M., Keniya M.V., Monk B.C. (2023). Functional Expression of Recombinant *Candida auris* Proteins in Saccharomyces cerevisiae Enables Azole Susceptibility Evaluation and Drug Discovery. J. Fungi.

[50] Kean R., Ramage G. (2019). Combined Antifungal Resistance and Biofilm Tolerance: The Global Threat of *Candida auris*. mSphere.

[51] Melinte V., Tudor A.D., Bujoi A.G., Radu M.A., Văcăriou M.C., Cismaru I.M., Holban T.S., Mîrzan C.L., Popescu R., Ciupan R.C. (2024). *Candida auris* Outbreak in a Multidisciplinary Hospital in Romania during the Post-Pandemic Era: Potential Solutions and Challenges in Surveillance and Epidemiological Control. Antibiotics.

[52] Cancino-Muñoz I., Mulet-Bayona J.V., Salvador-García C., Tormo-Palop N., Guna R., Gimeno-Cardona C., González-Candelas F. (2025). Short-term evolution and dispersal patterns of fluconazole-resistance in *Candida auris* clade III. mBio.

[53] Daniela Bădiceanu C., Camelia Nuță D., Missir A., vasile Hrubaru M., Delcaru C., Mara Dițu L., Chifiriuc M., Limban C. (2018). Synthesis, structural, phisico-chemical characterization and antimicrobial activity screening of new thiourea derivatives. Farm. J..

[54] Daniela Bădiceanu C., Camelia Nuță D., Missir A., vasile Hrubaru M., Delcaru C., Mara Dițu L., Chifiriuc M., Limban C. (2018). New Derivatives of 2-Thiophene Carboxylic Acid: Synthesis, Structure and Antimicrobial Studies. Farmacia.

[55] Fierascu I., Fierascu I.C., Dinu-Pirvu C.E., Fierascu R.C., Anuta V., Velescu B.S., Jinga M., Jinga V. (2019). A Short Overview of Recent Developments on Antimicrobial Coatings Based on Phytosynthesized Metal Nanoparticles. Coatings.

[56] Fernandes L., Ribeiro R., Costa R., Henriques M., Rodrigues M.E. (2022). Essential Oils as a Good Weapon against Drug-Resistant *Candida auris*. Antibiotics.

[57] Ríos-López A.L., Muñiz-Bernal V., Dávila-Aviña J., González G.M., Treviño-Rangel R.D.J., Becerril-García M.A., Flores-Maldonado O. (2024). Antifungal and antivirulence activity of tannic acid against drug-resistant *Candida* species. Farmacia.

[58] Arslan H., Duran N., Borekci G., Koray Ozer C., Akbay C. (2009). Antimicrobial Activity of Some Thiourea Derivatives and Their Nickel and Copper Complexes. Molecules.

[59] Moualek I., Iratni Aiche G., Mestar Guechaoui N., Lahcene S., Houali K. (2016). Antioxidant and anti-inflammatory activities of Arbutus unedo aqueous extract. Asian Pac. J. Trop. Biomed..

[60] Borman A.M., Fraser M., Johnson E.M. (2021). CHROMagarTM Candida Plus: A novel chromogenic agar that permits the rapid identification of *Candida auris*. Med. Mycol..

[61] Procop G.W. (2022). CLSI M27M44S.

[62] Chifiriuc C., Mihăescu G., Lazăr V. (2015). Microbiologie și Virologie Medicala.

[63] Lazăr V., Herelea V., Cernat R., Balotescu M.C., Bulai D., Moraru A. (2004). Microbiologie Generală: Manual de Lucrări Practice.

[64] Madhu G., Bose V.C., Aiswaryaraj A.S., Maniammal K., Biju V. (2013). Defect dependent antioxidant activity of nanostructured nickel oxide synthesized through a novel chemical method. Colloids Surf. A Physicochem. Eng. Asp..

[65] Kuroda K., Caputo G.A., DeGrado W.F. (2008). The role of hydrophobicity in the antimicrobial and hemolytic activities of polymethacrylate derivatives. Chem.-Eur. J..

[66] Dzah C.S., Zhang H., Gobe V., Asante-Donyinah D., Duan Y. (2024). Anti- and pro-oxidant properties of polyphenols and their role in modulating glutathione synthesis, activity and cellular redox potential: Potential synergies for disease management. Adv. Redox Res..

[67] Sotler R., Poljšak B., Dahmane R., Jukić T., Pavan Jukić D., Rotim C., Trebše P., Starc A. (2019). Prooxidant activities of antioxidants and their impact on health. Acta Clin. Croat..

[68] Gonzalez-Jimenez I., Perlin D.S., Shor E. (2023). Reactive oxidant species induced by antifungal drugs: Identity, origins, functions, and connection to stress-induced cell death. Front. Cell. Infect. Microbiol..

[69] Multescu M., Marinas I.C., Susman I.E., Belc N. (2022). Byproducts (Flour, Meals, and Groats) from the Vegetable Oil Industry as a Potential Source of Antioxidants. Foods.

[70] Bostiog D.I., Simionescu N., Coroaba A., Marinas I.C., Chifiriuc M.C., Gradisteanu Pircalabioru G., Maier S.S., Pinteala M. (2024). Multi-shell gold nanoparticles functionalized with methotrexate: A novel nanotherapeutic approach for improved antitumoral and antioxidant activity and enhanced biocompatibility. Drug Deliv..

[71] Purwantiningsih T.I. (2024). Antibacterial activity of Faloak (*Sterculia quadrifida* r. br) bark: A promising natural antibacterial candidate. Farmacia.

[72] (2018). M44: Method for Antifungal Disk Diffusion Susceptibility Testing of Yeasts.

[73] Corbu V.M., Gheorghe I., Marinaș I.C., Geană E.I., Moza M.I., Csutak O., Chifiriuc M.C. (2021). Demonstration of *Allium sativum* Extract Inhibitory Effect on Biodeteriogenic Microbial Strain Growth, Biofilm Development, and Enzymatic and Organic Acid Production. Molecules.

[74] Corbu V.M., Georgescu A.M., Marinas I.C., Pericleanu R., Mogos D.V., Dumbravă A.Ș., Marinescu L., Pecete I., Vassu-Dimov T., Czobor Barbu I. (2024). Phenotypic and Genotypic Characterization of Resistance and Virulence Markers in *Candida* spp. Isolated from Community-Acquired Infections in Bucharest, and the Impact of AgNPs on the Highly Resistant Isolates. J. Fungi.

[75] Marinas I.C., Ignat L., Maurușa I.E., Gaboreanu M.D., Adina C., Popa M., Chifiriuc M.C., Angheloiu M., Georgescu M., Iacobescu A. (2024). Insights into the physico-chemical and biological characterization of sodium lignosulfonate-silver nanosystems designed for wound management. Heliyon.

[76] Geana E.I., Ciucure C.T., Tamaian R., Marinas I.C., Gaboreanu D.M., Stan M., Chitescu C.L. (2023). Antioxidant and Wound Healing Bioactive Potential of Extracts Obtained from Bark and Needles of Softwood Species. Antioxidants.

